# Impact of surgeon-performed ultrasound on diagnosis of abdominal pain

**DOI:** 10.1136/emj.2007.052142

**Published:** 2008-07-25

**Authors:** A Lindelius, S Törngren, A Sondén, H Pettersson, J Adami

**Affiliations:** 1 Karolinska Institutet, Department of Clinical Science and Education, Södersjukhuset, Stockholm, Sweden; 2 Department of Surgery, Stockholm South General Hospital, Stockholm, Sweden; 3 Karolinska Institutet, Department of Medicine, Clinical Epidemiology Unit, Stockholm, Sweden

## Abstract

**Background:**

A randomised study was performed to evaluate the diagnostic accuracy of surgeon-performed ultrasound in the emergency department for patients presenting with abdominal pain.

**Methods:**

Surgeons responsible for the examination of study patients underwent 4 weeks of ultrasound training. 800 patients who were attending the emergency department for abdominal pain were randomised to undergo or not undergo surgeon-performed ultrasound as a complement to standard examination. The preliminary diagnosis made by the surgeon, with or without ultrasound, was compared with the final diagnosis made by a senior surgeon 6–8 weeks later.

**Results:**

Diagnostic accuracy was significantly higher in the group examined with ultrasound (64.7% vs 56.8%, p = 0.027). Ultrasound proved to be helpful in making or confirming a correct diagnosis in 24.1% of cases receiving ultrasound and to contribute in 2.9%. In 22.3% of patients the diagnosis of non-specific pain was confirmed by normal findings. Ultrasound was misleading in 10.2% of cases and had no influence on the diagnosis in 40.0%.

**Conclusion:**

For patients with acute abdominal pain, higher diagnostic accuracy is achieved when surgeons use ultrasound as a diagnostic complement to standard examination. The use of bedside ultrasound should be considered in emergency departments.

Abdominal pain is a common reason for seeking medical care in emergency departments all over the world.^1^ ^2^ Examination in the radiological department, including abdominal ultrasound (US), is performed in up to 65% of patients.^3^^–^5

In most countries US examinations are performed in radiology departments by specialised radiologists. The resources of the radiology departments are often limited, leading to long waiting times in the emergency department. Bedside US has been shown to reduce the length of stay in the emergency department, as well as the time to diagnosis and definitive care.^6^ ^7^ The use of bedside US performed by the surgeon or emergency physician is quite common in continental Europe and the USA.^8^^–^10 Several studies have confirmed the benefits of an early bedside US examination in trauma situations.^8^ ^11^^–^14 Studies evaluating diagnostic accuracy and other benefits of surgeon-performed US for patients with abdominal pain are fewer, but do exist.^15^^–^17 Nevertheless, the benefit of US examination performed by a surgeon in the emergency department for patients presenting with abdominal pain is still questioned.^8^ A search of the literature found a randomised study which evaluated diagnostic accuracy when radiologists performed immediate US on patients with abdominal pain.^18^ No randomised study evaluating the diagnostic accuracy of surgeon-performed US was found. We therefore designed a randomised clinical study to evaluate the effects of surgeon-performed US on patients admitted to the emergency department for abdominal pain.

## METHODS

### Setting

The study was conducted in the emergency department of Stockholm South General Hospital between February 2004 and June 2005. Stockholm South General Hospital is a public general hospital with 505 beds and a catchment area of about 600 000 inhabitants. The ED of Stockholm South General Hospital has an average of 100 000 visits per year by patients aged 15 years and older.

### Training of surgeons participating in the study

Nine surgeons, all with at least 2 years’ experience of surgery after completing an internship, attended a 1-week course led by a radiologist specialist in US. After attending the course they received 3 weeks of training in the radiological department in abdominal US under the guidance of a specialist in US. The surgeons were trained in detecting gallbladder stones, wide bile ducts, hydronephrosis, abdominal aortic aneurysms, ovarian cysts, free abdominal fluid, pleura fluid collections and large abdominal masses. They also had good knowledge about—and, in selected cases, were able to identify—an inflamed appendix, diverticulitis, intestinal obstruction, liver disease and large kidney stones. After completing the US training, the study surgeon worked in the emergency department for 4 weeks managing the study patients.

### Inclusion and exclusion criteria

All patients aged 18 years or older admitted to the emergency ward for abdominal pain were considered eligible to participate in the study. The exclusion criteria were pregnancy, previously diagnosed abdominal condition, acute conditions needing immediate care, inability to communicate with the investigator, drug or alcohol addiction and dementia.

### Baseline management

A total of 800 patients were enrolled for the study. After inclusion, the patients were examined by the study surgeon. Medical history was taken and clinical examination and routine laboratory testing were performed.

### Randomisation

After receiving the laboratory results, the study surgeon set a first preliminary diagnosis on a form containing 36 different predefined diagnoses. The form was then put in a sealed envelope. The sealed randomisation envelope was then opened and the patient was randomised to surgeon-performed US or no surgeon-performed US.

### Intervention

US examination was performed with one of two handheld 2.5–5 MHz or 4.3–6 MHz curved array transducers (Hawk 2102, transducers type 8801 and 8802, B-K Medical, Denmark) screening the entire abdomen. After US had been performed, the study surgeon completed a form with the results of the examination and gave a second preliminary diagnosis.

### Further management

The two groups were subsequently managed according to clinical routine as decided by the study surgeon. In both groups it was possible to request abdominal US from the radiological department, as well as complementary radiological examinations and blood tests if necessary, to provide secure medical care.

### Follow-up and collection of data

All information on the patients collected in the emergency department was entered by the study surgeon on a case report form. Additional data about patients admitted to the hospital for inpatient care were collected from the patient records and entered on a complementary case report form designed for the admission period.

After discharge from the emergency department or hospital ward, all patients were contacted by telephone by a study nurse 4–6 weeks after their first visit. The study nurse performed a structured interview including questions on state of health, performed and planned examinations, admission to other healthcare units and the patient’s evaluation of the visit to the emergency department.

### Outcomes, definition and measurement

#### Correct diagnosis

The correct diagnosis was defined as the final diagnosis given by a senior surgeon 6–8 weeks after the patient had entered the study, based on information in the patient records. When determining the final diagnosis, the senior surgeon was not aware of the preliminary diagnosis set by the surgeon in the emergency department.

The final diagnosis was compared with the preliminary diagnosis given in the emergency department, with or without US examination. The primary outcome of the study was defined as the proportion of correct diagnoses given in the emergency department.

#### Contribution of US to the diagnosis

The diagnoses of the patients in the US group were further analysed to elucidate the way in which US had contributed. Five groups with different contributions were defined:

1. US had contributed to the diagnosis either by changing an earlier incorrect diagnosis to a correct diagnosis or by confirming an earlier correct diagnosis.
2. US was misleading, either by confirming an earlier incorrect diagnosis or by changing an earlier diagnosis (correct or other incorrect) to another incorrect diagnosis. All cases where the surgeon had missed or incorrectly set the diagnosis of any of the conditions that he or she was supposed to diagnose according to the goal of education was defined as misleading. If the surgeon had changed to or confirmed an incorrect diagnosis by US, it was defined as misleading even if the final diagnosis was not included in the education goal.
3. US had no influence on making the diagnosis.
4. Non-specific abdominal pain (NSAP) was confirmed after US was performed. In this group US contributed to securing the diagnosis by a US examination that did not show any pathological findings leading to another diagnosis. In this group, NSAP was a correct diagnosis.
5. No correct diagnosis was made in the emergency department but US contributed to a correct diagnosis that was made later.

### Sample size

The sample size was calculated using SamplePower 2.0 based on the results from a previous prospective study^16^ and was set to detect a difference of nine percentage points in the proportion of correct diagnoses between the control and US groups (specifically 70% vs 79%). To detect this difference with 80% power at the 5% significance level (two-tailed), 400 patients were needed in each group.

### Statistical analysis

The χ^2^ test was used to compare the proportion of correct diagnoses between the intervention group and the control group (ie, the primary outcome measure); two-tailed p values of <0.05 were regarded as significant. The corresponding 95% confidence intervals (CI) for the difference between the proportions are based on the normal approximation. All analyses were performed according to intention-to-treat (ITT) and per protocol. SPSS V.14.0 was used for statistical analysis.

## RESULTS

**Figure 1:**
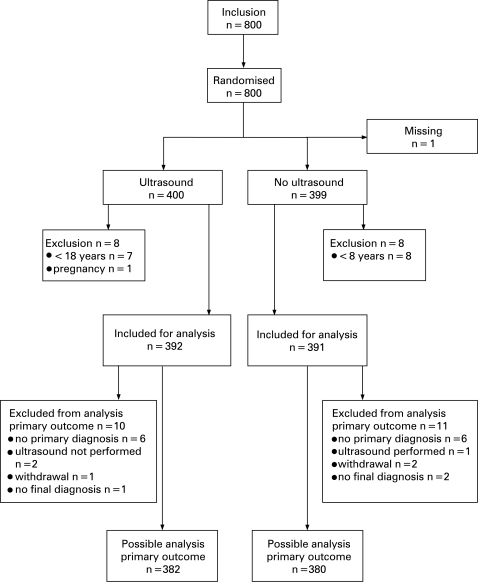
Flow chart of randomisation and follow-up.

Of the 800 patients randomised in the study, data on one patient were missing due to loss of the case report form and eight patients in each group failed to meet the inclusion criteria, leaving 392 patients in the US group and 391 patients in the control group eligible for statistical analysis. Data for evaluating the primary hypothesis (relation between primary diagnosis and final diagnosis) were missing in 10 of the 392 in the US group and 11 of the 391 in the control group, leaving 382/380 for this specific analysis (fig 1).

### Baseline characteristics

The groups were similar for all background factors except for referral pattern. More patients were referred from physicians in other specialties in the control group not undergoing US than in the US group (table 1).

**Table 1:** Baseline characteristics of study patients with abdominal pain at the emergency department

Characteristics	US (n = 392)		No US (n = 391)	
	Mean (SD)	n (%)	Mean (SD)	n (%)
Age	47 (20)		48 (19)	
Sex				
Male		160 (40.8)		171 (43.7)
Female		232 (59.2)		220 (56.3)
Length	172 (9)		172 (10)	
Weight	73 (16)		73 (16)	
Body mass index	24.8 (4.5)		24.8 (4.3)	
Abdominal-related co-morbidity		76 (19.4)		78 (19.9)
Co-morbidity related to heart or diabetes		66 (16.8)		74 (18.9)
History of abdominal malignancy		6 (1.5)		12 (3.1)
History of other malignancy		11 (2.8)		14 (3.6)
Other co-morbidity		132 (33.7)		123 (31.5)
Admission for abdominal pain within1 year		124 (32.0)		137 (35.3)
Referral for admission		92 (24.4)		126 (32.9)
Duration of pain				
0–8 h		44 (14.8)		43 (14.4)
8–24 h		99 (33.2)		97 (32.4)
>24 h		147 (49.3)		151 (50.5)
Cannot answer		8 (2.7)		8 (2.7)
Actual VAS (of pain)	4.3 (2.8)		4.4 (2.6)	
Maximal recall VAS (of pain)	7.6 (2.6)		7.6 (1.8)	
Temperature	37.0 (0.8)		37.0 (0.7)	
Affected general condition		90 (23.3)		74 (19.1)
Tenderness		338 (86.4)		347 (89.2)
Rigidity		51 (13.1)		49 (12.6)
Palpable mass		23 (5.9)		29 (7.5)

US, ultrasound; VAS, visual analogue scale (scale 0–10 where 0 represents no pain at all and 10 represents unbearable pain).

### Diagnostic accuracy

The proportion of correct primary diagnoses was 7.9 percentage points higher in the group undergoing US than in the control group (64.7% vs 56.8%; p = 0.027, 95% CI 0.01 to 0.15). Further analysis of the excluded patients did not change this result (table 2). The mean time between first diagnosis and diagnosis after US was performed was 23 min.

**Table 2:** Frequency of diagnostic accuracy and values in patients in the ultrasound (US) and no ultrasound (control) groups

	US, n (%)(n = 382)	No US, n (%)(n = 380)	Difference(%)	p Value
Correct diagnosis	247 (64.7)	216 (56.8)	7.9	0.027
				
Intention-to-treat (ITT) analyses	US (n = 400)	No US (n = 399)		
All excluded incorrect	247 (61.8)	216 (54.1)	7.7	0.029
All excluded correct	265 (66.3)	235 (58.9)	8.4	0.032
US correct, not US incorrect	265 (66.3)	216 (54.1)	12.2	<0.001
US incorrect, not US correct	247 (61.8)	235 (58.9)	2.9	0.410

### Contribution of US to correct diagnosis

In table 3 the contribution of US to the diagnosis is shown. US was helpful in making or confirming a specific diagnosis in 24.1%. In 22.5% of patients NSAP was confirmed by a normal US, and in 2.9% of patients the US findings contributed to making a correct diagnosis but the correct diagnosis was made after leaving the emergency department. US therefore helped in making the diagnosis in 49.5% of patients. In 10.2% of the patients US was considered misleading. Of these, 3 patients had a specified correct diagnosis changed into an incorrect one, in 8 patients a correct diagnosis of NSAP was changed into an incorrect diagnosis, in 7 patients a specified incorrect diagnosis was confirmed by US and in 20 patients US was misleading in another way. US had no influence on the diagnosis in 39.8% of cases and data were missing for two patients (table 3).

**Table 3:** Contribution of ultrasound (US) to diagnosis (n = 382)

	n (%)
Led to or confirmed correct diagnosis	92 (24.1)
Misleading (led to or confirmed an incorrect diagnosis)	39 (10.2)
No influence on diagnosis	153 (40.0)
Confirmed non-specific abdominal pain by normal findings	85 (22.3)
US contributed when further diagnoses were made but incorrect diagnosis made	11 (2.9)
Missing data	2 (0.5)
Total	382 (100.0)

### Final diagnoses

The final diagnoses made by the senior surgeon are shown in table 4. The most common diagnosis was NSAP which was made in approximately the same proportion in both groups. Other diagnoses differed to some extent between the groups, especially diverticulitis for which there were twice as many diagnoses in the group not undergoing US (table 4).

**Table 4:** Final diagnoses made by the senior surgeon for study patients with abdominal pain in the emergency department in the ultrasound (US) and no ultrasound (control) groups

	US, n (%)(n = 392)	No US, n (%)(n = 391)	Total(n = 783)
Non-specific abdominal pain	148 (37.9)	148 (38.1)	296 (38.0)
Cholelithiasis (symptomatic, with or without cholecystitis)	34 (8.7)	27 (7.0)	61 (7.8)
Appendicitis	34 (8.7)	29 (7.5)	63 (8.1)
Diverticulitis	17 (4.3)	35 (9.0)	52 (6.7)
Ureteric calculus (symptomatic, with or without hydronephrosis)	22 (5.6)	23 (5.9)	45 (5.8)
Urinary tract infection (cystitis or pyelonephritis)	18 (4.6)	14 (3.6)	32 (4.1)
Dyspepsia/reflux/oesophagitis	16 (4.1)	12 (3.1)	28 (3.6)
Gastroenteritis/virosis	14 (3.6)	10 (2.6)	24 (3.1)
Choledocholithiasis (with or without cholangitis or pancreatitis)	9 (2.3)	13 (3.4)	22 (2.8)
Tumour (not previously known)	10 (2.6)	11 (2.8)	21 (2.7)
Ovarian cyst (symptomatic, with			
or without rupture/bleeding)	7 (1.8)	11 (2.8)	18 (2.3)
Pancreatitis (without calculus)	11 (2.8)	5 (1.3)	16 (2.1)
Muscle-related pain	9 (2.3)	5 (1.3)	14 (1.8)
Ileus/subileus	3 (0.8)	10 (2.6)	13 (1.7)
Constipation/faecaloma	6 (1.5)	4 (1.0)	10 (1.3)
Hydronephrosis (without ureteric calculus)	4 (1.0)	3 (0.8)	7 (0.9)
Abscess	4 (1.0)	3 (0.8)	7 (0.9)
Salpingitis	6 (1.5)	1 (0.3)	7 (0.9)
Hernia	5 (1.3)	1 (0.3)	6 (0.8)
Colitis/terminal ileitis	0 (0.0)	5 (1.3)	5 (0.6)
Duodenal ulcer without perforation	2 (0.5)	0 (0.0)	2 (0.3)
Perforated duodenal ulcer	1 (0.3)	1 (0.3)	2 (0.3)
Abdominal aortic aneurysm	0 (0.0)	1 (0.3)	1 (0.1)
Other diagnosis	11 (2.9)	16 (4.1)	27 (3.4)

## DISCUSSION

In this large randomised study which assessed the use of US for diagnosing abdominal pain, a small but significant increase was seen in the frequency of correct diagnoses in patients undergoing surgeon-performed US.

The number of patients seeking medical care in the emergency department for abdominal pain is high. There is often a need for complementary radiological examinations in this group of patients, and waiting times for these examinations are often long. As more acute radiological examinations are performed, the waiting time for an elective radiological examination is increasing. If it was possible for the surgeon or emergency physician to perform bedside US, this might decrease the need for complementary radiological examinations and thereby give the radiologists more time for performing elective and specialised examinations.

One of the strengths of this study is the large number of patients, with very few refusals and complete follow-up for the preliminary outcome. One possible weakness of the study design is that the examinations were not validated by a US examination performed by a radiologist. However, it was our intention that the study design should reflect possible real situations.

The final diagnoses differed to some extent, with a diagnosis of diverticulitis being twice as frequent in the patients who did not undergo US examination. The reported diagnostic accuracy for diverticulitis on a clinical basis varies, but it is not in the lowest range of diagnostic accuracy.^4^ ^16^ ^19^ ^20^ This difference would therefore probably not influence the results of our study.

Apart from the US examination itself, the reason for the higher number of correct diagnoses in the US group could be the fact that these patients had one additional examination since it is natural to palpate the patient’s abdomen and speak to the patient when performing US. Nevertheless, in this study we wanted to examine the real situation in the emergency department, and this additional time with the patient would, of course, also be the situation in clinical practice.

An earlier study has shown that bedside US has a significant impact on diagnostic certainty.^21^ Our study has shown that US examination contributes, not only by leading to a correct diagnosis but also by confirming an already correct diagnosis and thereby increasing diagnostic certainty. The number of patients in whom US was misleading was quite high but substantially smaller than the proportion in whom US was helpful. These numbers were similar—although in the higher range—to the results from other studies evaluating the contribution of early US performed by a radiologist, where the proportion of examinations leading to an incorrect diagnosis ranged between 2% and 11%.^18^ ^22^^–^24 Of the 10.2% misleading cases, only in three cases was a correct specific diagnosis changed to an incorrect diagnosis and in eight cases a correct diagnosis of NSAP was changed to an incorrect diagnosis. This is a small group but should be considered in the overall picture if US is implemented. However, we believe that the higher number of correct diagnoses with US could justify this group of misclassified patients.

Education of the surgeon or emergency physician performing US is of great importance and has been widely debated.^25^ ^26^ Guidelines produced by the Society for Academic Emergency Medicine (SAEM) in 1994 called for a minimum of 150 total US examinations and 40 h of didactic instruction, including gynaecological and cardiovascular US training.^25^ ^26^ The US training in our department includes 40 h of didactic training and 120 h of abdominal US scanning on patients referred for US, all evaluated by a specialist in US. We think that this training is comparable to that recommended in the SAEM guidelines and consider that the knowledge in basic US achieved by the surgeons is reliable, even though there was no regular examination at the end of the training period. It is important to realise that, when surgeons and emergency physicians perform US in the emergency department, it is used as a complement to patient history, physical examination and laboratory tests for making a diagnosis and deciding about further management. Education and training for this purpose will differ from that of the radiologist, whose role as a specialist performing US with specific aims will still be of great importance for diagnosing patients as well as for intervention.

## CONCLUSION

This study shows that surgeon-performed US significantly increases diagnostic accuracy in patients with abdominal pain attending the emergency department. It is concluded that US is a helpful diagnostic tool in these patients and the use of bedside US in the emergency department is recommended.
